# Wound healing with topical BRAF inhibitor therapy in a diabetic model suggests tissue regenerative effects

**DOI:** 10.1371/journal.pone.0252597

**Published:** 2021-06-23

**Authors:** Helena Escuin-Ordinas, Yining Liu, Lu Sun, Willy Hugo, Robert Dimatteo, Rong Rong Huang, Paige Krystofinski, Ariel Azhdam, Jordan Lee, Begoña Comin-Anduix, Alistair J. Cochran, Roger S. Lo, Tatiana Segura, Philip O. Scumpia, Antoni Ribas

**Affiliations:** 1 Division of Hematology/Oncology, Department of Medicine, University of California, Los Angeles (UCLA), Los Angeles, California, United States of America; 2 Department of Chemical and Biomolecular Engineering, UCLA, Los Angeles, California, United States of America; 3 Division of Dermatology, Department of Medicine, UCLA, Los Angeles, California, United States of America; 4 Department of Pathology and Laboratory Medicine, UCLA, Los Angeles, California, United States of America; 5 Department of Dermatology, VA Greater Los Angeles Healthcare System-West Los Angeles, Los Angeles, California, United States of America; 6 Division of Surgical Oncology, Department of Surgery, UCLA, Los Angeles, California, United States of America; 7 Department of Molecular and Medical Pharmacology, UCLA, Los Angeles, California, United States of America; 8 Jonsson Comprehensive Cancer Center, UCLA, Los Angeles, California, United States of America; 9 Department of Biomedical Engineering, Duke University, Durham, North Carolina, United States of America; 10 Department of Biological Chemistry, UCLA, Los Angeles, California, United States of America; Rutgers University, UNITED STATES

## Abstract

Wound healing is a multi-step process to rapidly restore the barrier function. This process is often impaired in diabetic patients resulting in chronic wounds and amputation. We previously found that paradoxical activation of the mitogen-activated protein kinase (MAPK) pathway via topical administration of the BRAF inhibitor vemurafenib accelerates wound healing by activating keratinocyte proliferation and reepithelialization pathways in healthy mice. Herein, we investigated whether this wound healing acceleration also occurs in impaired diabetic wounds and found that topical vemurafenib not only improves wound healing in a murine diabetic wound model but unexpectedly promotes hair follicle regeneration. Hair follicles expressing Sox-9 and K15 surrounded by CD34+ stroma were found in wounds of diabetic and non-diabetic mice, and their formation can be prevented by blocking downstream MEK signaling. Thus, topically applied BRAF inhibitors may accelerate wound healing, and promote the restoration of improved skin architecture in both normal and impaired wounds.

## Introduction

Truly an epidemic, the number of people with diabetes mellitus has quadrupled in the past three decades with 1 in 11 adults worldwide now having diabetes mellitus, 90% of whom are type 2 diabetes mellitus (T2DM). Despite increasing awareness, a number of factors including poor vascular flow and sensory neuropathy in these patients impede proper wound healing leading to chronic wounds that often heal poorly, if at all, culminating in subsequent limb amputation in up to 15% of diabetic wounds [1]. The cost to treat diabetic foot ulcers is estimated at up to 13 billion dollars annually and despite use of expensive advanced wound care devices, these wounds recur in up to 50% of patients [2, 3]. Clearly, new regenerative approaches to accelerate diabetic wound healing and restore tissue structure and function to better withstand reinjury in diabetic wounds is necessary.

The Ras/MEKK/MEK/ERK pathway is activated following cutaneous injury [4] and regulates DNA synthesis, cell proliferation, and cellular migration [5]. Pharmacologic activation of ERK can promote wound healing responses [6], but how this pathway participates in wound healing and tissue structure repair in impaired, poorly healing wounds is currently unknown. Paradoxical MAPK activation, where the preferential binding of the V600-mutant specific BRAF inhibitor to a BRAF protomer results in the transactivation of its CRAF heterodimer partner, is a well-known property of BRAF inhibitors [7–11]. In our previous study, we showed that topical administration of BRAF inhibitors triggers paradoxical activation on keratinocytes, driving MEK/ERK activation, increased MAPK transcriptional output, enhanced cell migration and proliferation which results in accelerated wound healing [12]. In this study, we extend these findings to show that topical V600-mutant specific BRAF inhibitors promote wound healing in a db/db mouse model of impaired diabetic wound healing. Remarkably, in both the diabetic and non-diabetic models [12], vemurafenib induced regenerative wound healing characterized by diminished scar tissue with hair follicle formation within the wounds. Mechanistically, vemurafenib treatment allowed for β-catenin activation, and treatment with the MEK inhibitor trametinib prevented hair follicle formation while limiting β-catenin activation. These findings indicate that paradoxical MAPK activation by topical vemurafenib application can not only accelerate wound healing and induce skin regeneration in impaired wounds but also highlight a previously unrecognized potential to regenerate functional skin [13, 14].

## Materials and methods

### Ethics statement

The mouse studies described in this manuscript were performed under the written approval of the UCLA Animal Research Committee (ARC) in accordance with all federal, state, and local guidelines. All studies were carried out under strict accordance with the guidelines in The Guide for the Care and Use of Laboratory Animals of the National Institutes of Health and the accreditation and guidelines of the Association for the Assessment and Accreditation of Laboratory Animal Care (AALAC). The protocol/permit/project license number assigned by the IACUC/ethics committee is UCLA ARC Protocol Number 2010-011-13J or 2013-066-01. Wound healing experiments were performed under isoflurane anesthesia and all efforts were made to minimize animal pain and discomfort.

### Western blotting

Human adult epidermal keratinocytes (HEKa, purchased from Invitrogen (Grand Island, NY; C-005-5C)) were treated in duplicate with a concentration of 1.5 μM of vemurafenib or vehicle control. Primary antibodies included p-ERK Thr204/205 (1:1000, #9101), ERK (1:1000, #4094) and GAPDH (1:3500, #5174), (all from Cell Signaling Technology, Danvers, MA); IGFBP3 (1:1000, #10189-2AP; Proteintech, Rosemont, IL) and Anti-Frizzled-7 (5ug/ml, #ab64636; Abcam, Cambridge, MA). Immuno-reactivity was revealed with an ECL-Plus kit Amersham Biosciences Co, Piscataway, NJ), using a Chemidoc scanner (BioRad laboratories, Hercules, CA).

### Excisional skin wound splinting model

Seven to nine-week-old female db/db mice were used for these studies under the ACR protocol #2010-011-13F. Mice were anesthetized with 2–3% isoflurane in an induction chamber and kept under anesthesia during the whole surgery. The back of the mice was shaved, sterilized with betadine and 70% ethanol. A dose of buprenorphine (2.5 mg/kg) was administered subcutaneously before the surgery. Two excisional wounds were made in the skin aside from the midline of the animal using a 6-mm biopsy punch. 20 μl of vemurafenib (0.1 mg/μl) in DMSO or DMSO was applied topically on the wounds one minute before suturing the splinting rings. The splinting rings have an 8-mm transparent window, which was covered with Tegaderm to allow visualization and measurement of the wound size. All animals were observed for signs of inflammation and pain for the first 48 hours’ post-surgery. Vemurafenib or DMSO was repeatedly applied on day 2 and 4. Wounds were photographed at day 0, day 2, day 6 and day 14, based on which the percentages of wound closure were calculated. Seven to nine-week-old female Balb/c mice were treated as stated in the methods of our previous work [12].

### Incisional wound model

C3Hf/Kam (H2-k) female mice were used for wound-healing studies at 8–10 weeks of age. They were bred at UCLA and used under the Animal Research Council (ARC) protocol # 2013-066-01. Full-thickness wounds ∼2.5 cm long were made in the shaved dorsal skin of anesthetized mice as described [15], with ketamine/xylazine as an anesthetic. Clinical grade vemurafenib or trametinib pills (Zelboraf, Genentech, South San Francisco, CA, USA) were grinded and dissolved in dimethylsulfoxide (DMSO; Fisher Scientific) and phosphate buffered saline (PBS; diluted 1:4) to a concentration of 40 μg/μl and 50 μl of the mixture (or DMSO in PBS as vehicle control) was added topically. Wounds were closed with 3–4 clips, which were removed after 2 days. Vemurafenib suspension (2 mg), with and without trametinib (0.2mg), or vehicle control was re-applied topically to the wound site on days 2, 4, 6, 8, 10 and 12. Samples were collected on days 6 and 14.

### Histological analysis

Dorsal skin wounds from db/db mice treated with vemurafenib suspension or DMSO in saline control suspension were sacrificed at day 2, 6 and 14 with isoflurane overdose and the wounds excised at those time point. Two 8-mm round pieces of tissue were collected from each Db/Db mouse containing the whole wound area and the surrounding tissue and skin, cut precisely in half at the midline of the wound and fixed in 1% paraformaldehyde (PFA) for 16–18 hours at 4°C, dehydrated in 70% EtOH, and then paraffin-embedded. Balb/c and CH3 mice wound treatment for histological analysis is described in our previous publication [12]. Sections were cut at 4 μm, deparaffinized with xylene and descendant ethanol, and then incubated in 3% H2O2 for 10 minutes. After a wash in distilled water, the slides were incubated for 25 minutes in citrate buffer pH 6 (Invitrogen, Carlsbad, CA) at 95°C using a vegetable steamer. The slides were brought to room temperature, rinsed in PBST (Phosphate Buffered Saline containing 0.05% Tween-20), and then incubated at room temperature with 1:100 Ki-67 (DAKO, Carpinteria, CA), 1:1000 PECAM-1 (Santa Cruz Biotechnology, Dallas, Texas) and 1:50 Frizzled-7 (Abcam, Cambridge, MA) for 1 hour and 1:10 phospho-ERK Ab (Cell Signaling Technology, Danvers, MA.), 1:800 Sox-9 (Abcam, Cambridge, MA), 1:100 non-phospho (active) β-catenin (Ser33/37/Thr41), 1:100 β-catenin (both β-catenin antibodies were purchased from Cell Signaling, Danvers, MA), 1:1000 K15 (Biolegend, San Diego, CA) and 1:2000 CD34 (Abcam, Cambridge, MA) overnight at 4°C. The stained slides were rinsed with PBST and incubated at room temperature with 1:200 polyclonal Rabbit anti-rat immunoglobulin/Biotinylated Ab (Dako, Carpinteria, CA #E0468) for 30 minutes. All slides were rinsed with PBST and incubated with Dako EnVision+ System–HRP Labelled Polymer Anti-Rabbit (Dako, Carpinteria, CA) at room temperature for 30 minutes. After a rinse with PBST, the slides were incubated with DAB (3,3’-Diaminobenzidine) for visualization. Subsequently, the slides were washed in tap water, counterstained with Harris’ Hematoxylin, dehydrated in ethanol, and mounted with media. The imaging and quantification of our cell-based immunohistochemistry was performed with the HALO Next Generation Imaging analysis software (Indica Labs; Corrales, NM). HALO measures and reports individual cell data maintaining an interactive link between cell metrics and cell imagery. The number of pERK+ Ki67+, PECAM-1+, Sox-9+, CD34+K15+ and Frizzled+ cells was automatically counted with the HALO software. Three 20x fields of view from each side of the wound were automatically counted for pERK, Ki67 and Sox-9 stains. PECAM-1+ cells were automatically counted on each side of the wound edges where the granulation tissue starts (1 mm length each side) on the excisional wound-splinting model, and in the entire wound area on the incisional wound model. Hair follicles within the wound area were counted with the HALO software on the H&E slides. CD34K15+ and Frizzled+ cells were automatically counted within the wound area. Wound closure scores were determined based on the percentage of the wound that has been re-epithelialized compared to the total wounded area based on the average of two sections of tissue. Scores are defined as 0: 0–25% re-epithelialized, 1: 25–50% re-epithelialized, 2: 50–75% re-epithelialized, 3: 75–99% re-epithelialized, and 4: completely re-epithelialized.

### RNAseq analysis

The entire wound area (epidermis/dermis) from formalin-fixed paraffin-embeded (FFPE) slides, treated with vehicle or vemurafenib, was cut with a laser using the Leica LMD7000 microdissection system and RNA was extracted (RNeasy Mini Kit, Qiagen, Valencia, CA) and sent for RNAseq analysis using 1x50bp single end read on Illumina HiSeq3000 (Illumina, San Diego, CA). Raw sequences were mapped to the mouse mm10 reference sequence by HISAT2 [16]. Gene level counts were generated by the program htseq-count [17] and expression normalization and differential gene expression analysis were done using DESeq2 [18]. Genes with 1.5-fold up-/downregulation of mRNA expression (computed based on the averages of the triplicates of each treatment group) were submitted to the online tool ENRICHR [19, 20].

### Statistical analysis

Data were analyzed with GraphPad Prism (version 5) software (GraphPad Software, La Jolla, CA). Significance was determined by unpaired two-tailed Student’s t-test or one-way analysis of variance (ANOVA). Variance was similar between the groups that were statistically compared. All measurements were taken from distinct samples.

### Code availability statement

The RNaseq pipeline was done as previously [12] and the authors agree to share the code upon request.

## Results

### Topical vemurafenib accelerates wound healing in a diabetic mouse model

**Fig 1:**
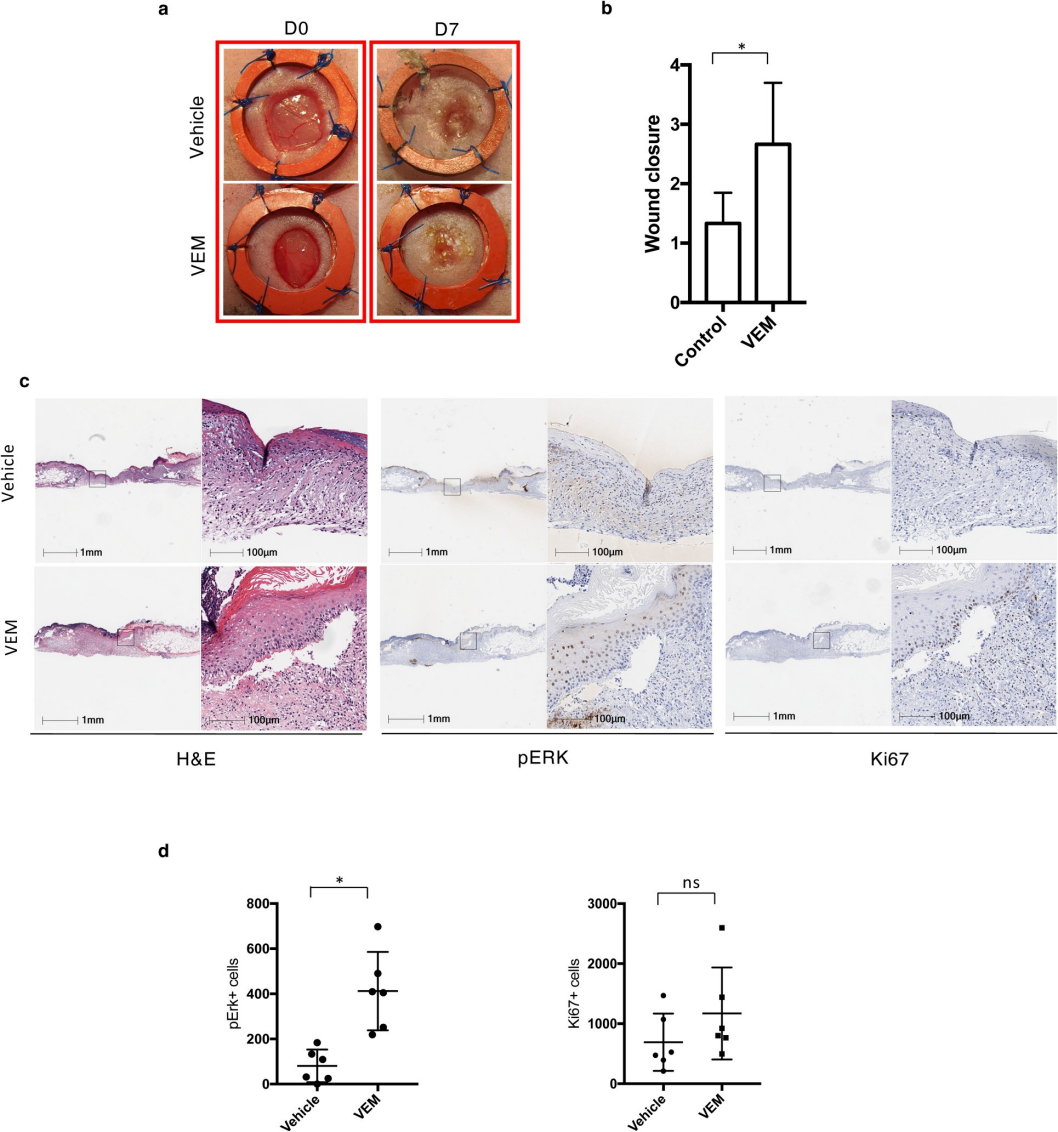
Topical BRAF inhibition accelerates wound healing in an excisional diabetic wound model. (a) Representative images of vehicle-treated and vemurafenib (vemurafenib)-treated wounds on day 0 and day 7. (b) Wound closure scores of vehicle-treated and vemurafenib-treated mice on day 0 and day 7 (*P = 0.04, n = 6). (c) Representative H&E and pERK and Ki67 staining IHC images in the presence and absence of vemurafenib by day 7. (d) Box plots representing the quantification of pERK^+^ (*P = 0.01) and Ki67^+^ (NS) cells, for control wounds versus treated wounds on day 7. NS, not significant; H&E, hematoxylin and eosin; IHC, immunohistochemistry. Error bars in b and d, mean ± s.d. Bar graphs represent one experiment with 6 replicates per group.

To test whether topical application of vemurafenib could accelerate wound healing in an impaired wound healing model, we performed an excisional wound splinting model in db/db mice [21], a model that demonstrates severe impairment in wound closure. We induced 6-mm round wounds on the back of mice; splinting rings were tightly adhered and sutured to the skin around the wounds, preventing wound closure caused by skin contraction. Vemurafenib (2 mg) or DMSO vehicle control was applied on the wounds on days 0, 2 and 4, and percent wound closure was sequentially measured. On Day 7, the wounds treated with vemurafenib showed a significantly higher wound closure score compared to the ones treated with vehicle control (p = 0.004, n = 6, by t-test; Fig 1A and 1B). The area of the wounds and their surroundings were analyzed histologically and pERK^+^ and Ki67^+^ cells were quantified using digital pathology (Fig 1C and 1D). The number of pERK^+^ cells in the wounds treated with vemurafenib was statistically significantly higher by day 7 compared to the control group (p = 0.01 by t-test; n = 6; Fig 1C and 1D), demonstrating paradoxical MAPK leading to enhanced wound closure in impaired diabetic wounds. The number of Ki67^+^ cells were also higher on the vemurafenib-treated mice compared to the control mice (ns by t-test; n = 6; Fig 1C and 1D). The healing process in the presence of vemurafenib was accelerated compared to controls, showing initiation of re-epithelialization on the edges of the wounds treated with vemurafenib by day 7 (Fig 1B).

**Fig 2:**
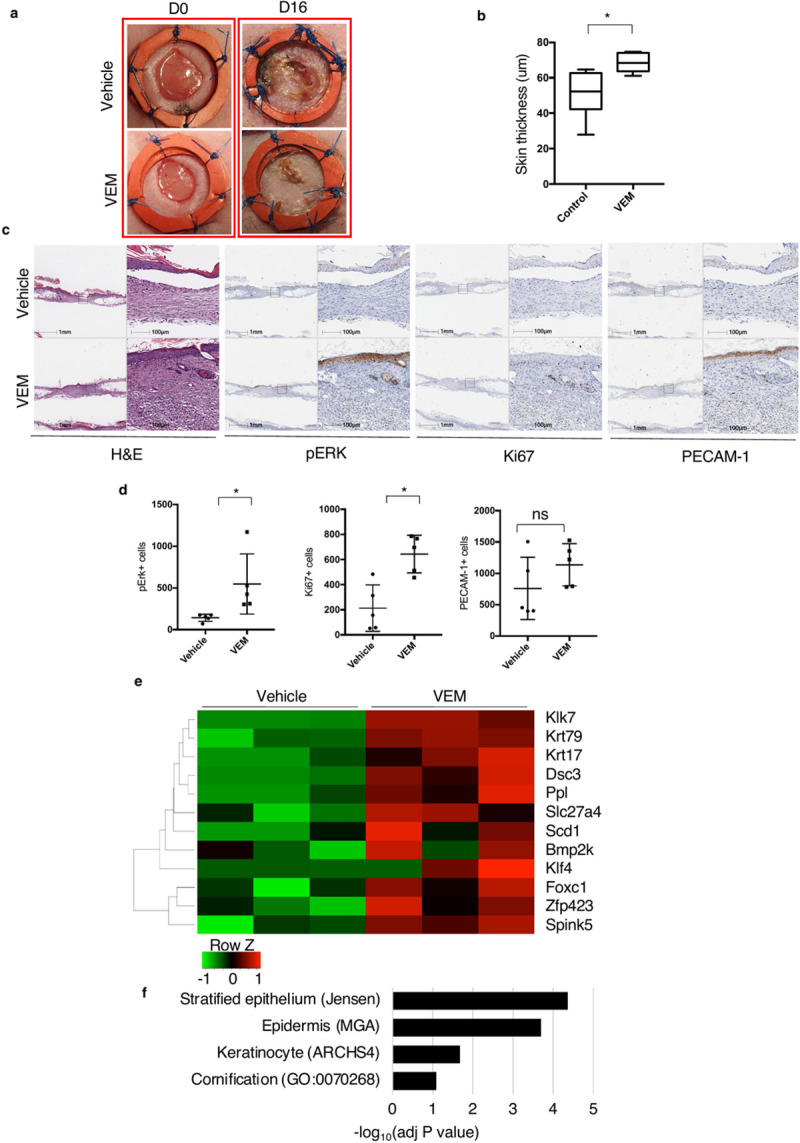
Topical vemurafenib promotes wound closure in diabetic wounds by Day 16. (a) Representative images of vehicle- and vemurafenib-treated wounds on day 0 and day 16. (b) Epidermal+dermal thickness quantification of vehicle- and vemurafenib-treated wounds by Day 16 (*P = 0.02). (c) Representative H&E, pERK and Ki67 staining IHC images in the presence and absence of vemurafenib by day 16. (d) Scatter plots representing the quantification of pErk+ (P = 0.04), Ki67 (*P = 0.015); for control wounds versus treated wounds on day 16. (e) Heatmap of representative genes that are differentially expressed either the vemurafenib-treated or control wounds on Day 16. (f) Pathway/gene ontology enrichment analyses of the full list of differentially expressed genes (at least 1.5-fold difference) using ENRICHR. Enriched gene sets (adjusted P ≤ 0.1, Jensen: Jensen tissues database, MGA: mouse gene atlas, ARCHS4: tissue RNAseq dataset, GO: gene ontology database) are listed. NS, not significant; H&E, hematoxylin and eosin; IHC, immunohistochemistry. Error bars in b and d, mean ± s.d. Bar graphs represent one experiment with 5 replicates per group.

While the re-epithelialization was complete earlier in VEM treated samples (by day 7), both groups showed complete re-epithelialization by Day 16. There was a clear difference in skin (epidermal + dermal) thickness between the control and the vemurafenib-treated group, the latter being significantly thicker (p = 0.02 by t-test; n = 5; Fig 2A and 2B). The level of pERK^+^ cells in the wounds treated with vemurafenib was also statistically significantly higher (p = 0.04 by t-test; n = 5), as well as the number of Ki67^+^ cells (p = 0.015 by t-test; n = 5), compared to the control group by day 16 (Fig 2C and 2D).

To further characterize these findings, samples obtained from the diabetic wound-healing model were analyzed by RNASeq. The list of differentially expressed genes on day 16 in samples obtained from db/db mice (selected genes in Fig 2E and 2F and full listing in S1B Table) i.e. genes that were differentially expressed by 1.5-fold or more in either the vemurafenib or DMSO treated group, were tested for gene set/ontology enrichment using ENRICHR [19, 20]. The vemurafenib-treated wounds showed significantly increased expression of mRNA genes related to keratinocyte differentiation, confirming that vemurafenib-treated wounds displayed more advanced re-epithelialization than those of DMSO-treated wounds. Of note, *Krt17* and *Krt79*, two markers used to track hair follicle epithelial cells, particularly those involved in morphogenesis, migration, and wound regeneration [22–24] were expressed at higher levels, suggesting enhanced keratinocyte differentiation towards appendageal structures in vemurafenib-treated wounds. Krt17 is also a well know proliferation marker for keratinocytes. Thus, its upregulation in VEM-treated wounds could also be the results of hyperproliferation due to activation of MAPK pathway.

### Topical vemurafenib promotes hair follicle regeneration in db/db wounds

**Fig 3:**
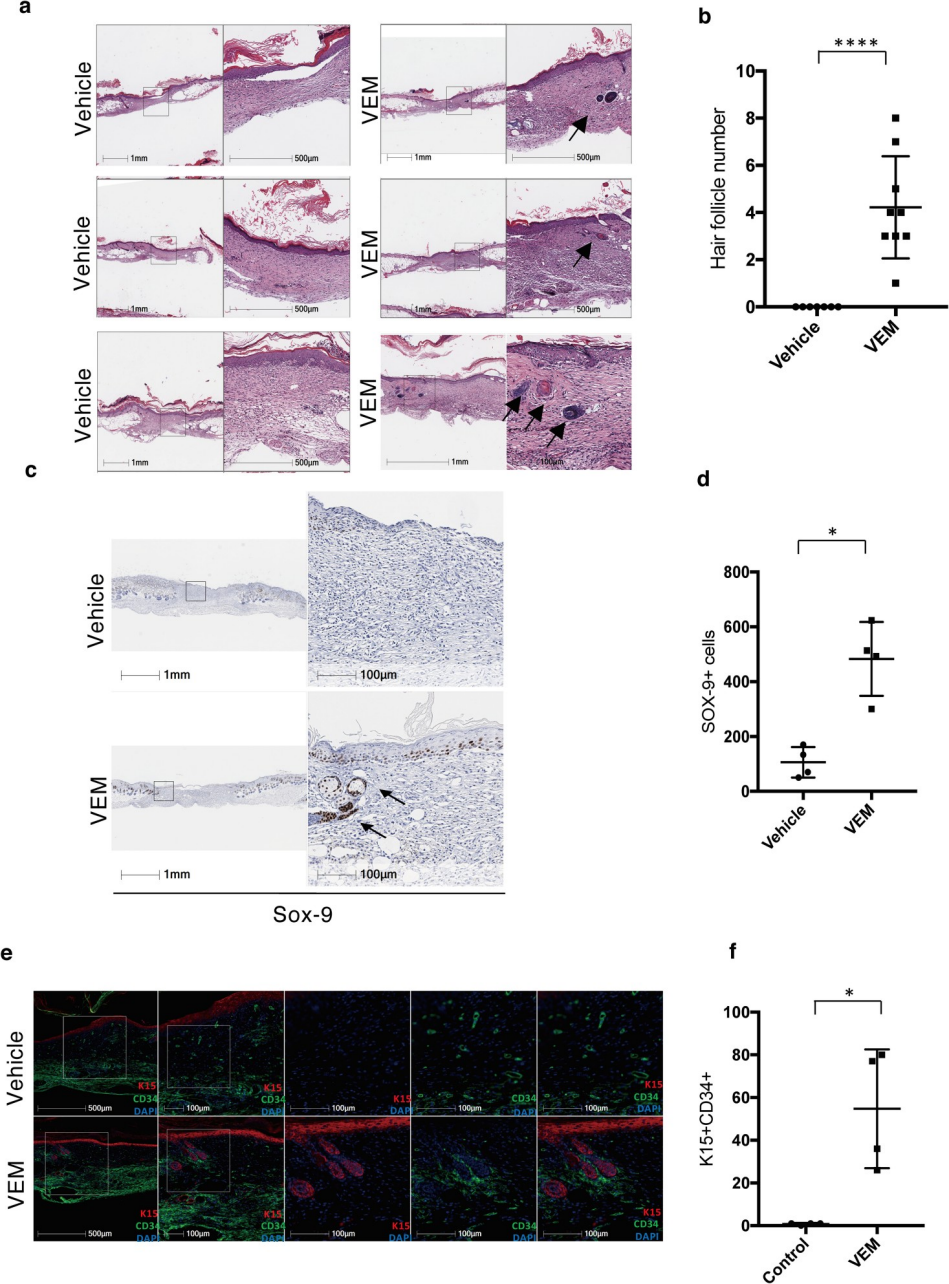
Topical vemurafenib promotes hair follicle regeneration in db/db wounds. (a) Representative H&E images of vehicle-treated and vemurafenib (vemurafenib)-treated wounds on day 0 and day 16 in diabetic mice. (b) Scatter plots representing the quantification of hair follicles within the wound area (****P = 0.0001) for control wounds versus treated wounds on day 16 in diabetic mice. (c) Representative Sox-9 staining IHC images in the presence and absence of vemurafenib in db/db mice wounds by day 16. (d) Scatter plot representing the quantification of Sox-9+ cells (*P = 0.02, n = 4) for control wounds versus treated wounds on day 16. (e) Representative CD34, K15 and DAPI staining IF images in the presence and absence of vemurafenib in db/db mice wounds by Day 16. (f) Scatter plots representing the quantification of CD34+ K15+ cells for control wounds versus treated wounds (*P = 0.02). Error bars in b, d and f mean ± s.d. Bar graphs represent one experiment with 4–9 replicates per group.

Upon close histologic examination of the healed wounds on day 16, wounds treated with vemurafenib displayed scattered hair follicle structures within the wound beds (Figs 2C and 3A). Many of these hair follicles had associated sebaceous glands, and they appeared disorganized, some emerging from within follicular cysts within the mid to upper dermis, unlike the larger, orderly hair follicles that embed in the subcutaneous tissue from the wound edge (Fig 3A). DMSO-treated wounds displayed typical scar appearance with flattened epidermis with parallel collagen bundles, and vertical blood vessels within a dermis devoid of adnexal structures (Figs 2C and 3A). This latter finding is not surprising as small wounds in otherwise normal mice heal with scar formation while larger wounds often heal with neogenic hair formation [15]. Histologic quantification confirmed the presence of hair follicles in all vemurafenib treated wounds, while all DMSO-treated wounds displayed scar formation with minimal to no hair follicle formation (Fig 3B).

Since Sox9 has been shown to mark hair follicle stem cells [25], we performed immunohistochemistry for Sox9 to confirmed the presence of Sox9 expressing cells in vemurafenib treated samples. Indeed, we found an increase in staining of follicular structures in the dermis in wounds of vemurafenib-treated db/db mice when compared to wounds of DMSO-treated db/db mice (Fig 3C and 3D). These results were confirmed by examining immunofluorescence of keratin15 and CD34, two additional markers of hair follicle/bulge cells (Fig 3E and 3F). Taken together, these findings suggest that topical vemurafenib can not only accelerate wound healing in impaired wounds but may stimulate regenerative healing in these wounds as well.

### Topical vemurafenib promotes regenerative healing in Balb/c wounds

**Fig 4:**
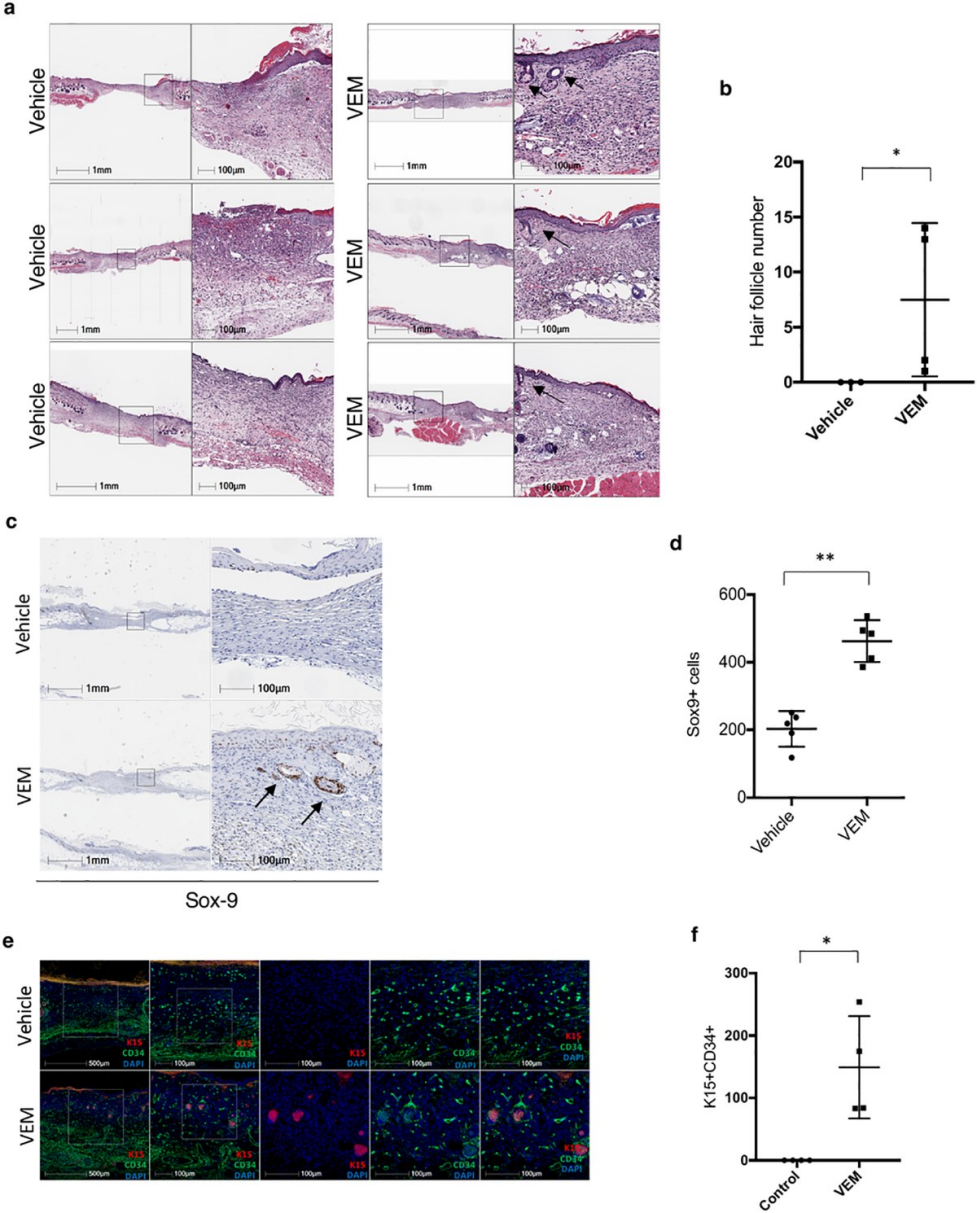
Topical vemurafenib promotes hair follicle regeneration in Balb/c wounds. (a) Representative H&E images of vehicle-treated and vemurafenib (vemurafenib)-treated wounds on day 0 and day 14 in Balb/c mice. (b) Scatter plots representing the quantification of hair follicles within the wound area (*P = 0.02) for control wounds versus treated wounds on day 14 in Balb/c mice. (c) Representative Sox-9 staining IHC images in the presence and absence of vemurafenib in Balb/c mice wounds by day 14. (d) Scatter plots representing the quantification of Sox-9+ cells (*P = 0.02, n = 4) for control wounds versus treated wounds on day 14. (e) Representative CD34, K15 and DAPI staining immunofluorescent images in the presence and absence of vemurafenib in Balb/c mice wounds by day 14. (f) Scatter plots representing the quantification of K15+ with surrounding CD34+ stroma for control wounds versus treated wounds (*P = 0.02). Error bars in b, d and f mean ± s.d. Bar graphs represent one experiment with 4–5 replicates per group.

We next wished to evaluate if this phenomenon of regenerative wound healing was a nuance of the db/db mouse model or could be generalized to other models. We previously used a non-impaired Balb/c mouse strain to evaluate the effects of vemurafenib on wound healing in a splinted model similar to the one we performed in diabetic mice above [12], but did not evaluate whether these wounds displayed evidence of hair follicle regeneration. Indeed, we observed the appearance of hair follicles on all the re-epithelialized wounds treated with vemurafenib by day 14, whereas the control wounds did not have any sign of hair follicle regeneration (Fig 4A). There was some variability in the Balb/c wounds, with some mice demonstrating high levels of hair follicle regeneration, while other displaying much lower numbers in response to vemurafenib (Fig 4B). The histologic appearance of these hair follicles was again of disorganized hairs. Several displayed connections to the epidermis, and their depth to the superficial to mid dermis within the wound bed were different than the hair follicles of the normal adjacent skin which were embedded in the fat. Similar to the db/db wounds, those in Balb/c wounds displayed strong staining with the hair follicle marker stem and progenitor markers Sox9 (Fig 4C), as well as KRT15 and CD34 (Fig 4E and 4F). The levels of SOX-9^+^ cells were significantly higher on the vemurafenib-treated Balb/c mice wounds compared to the control wounds (p = 0.02 by t test; n = 4; Fig 4D). These findings confirmed that hair follicle regeneration induced by topical vemurafenib can occur in otherwise healthy strains of mice, and in response to small wounds that typically do not result in regenerative healing.

### Topical vemurafenib activates Wnt-β-catenin signaling

**Fig 5:**
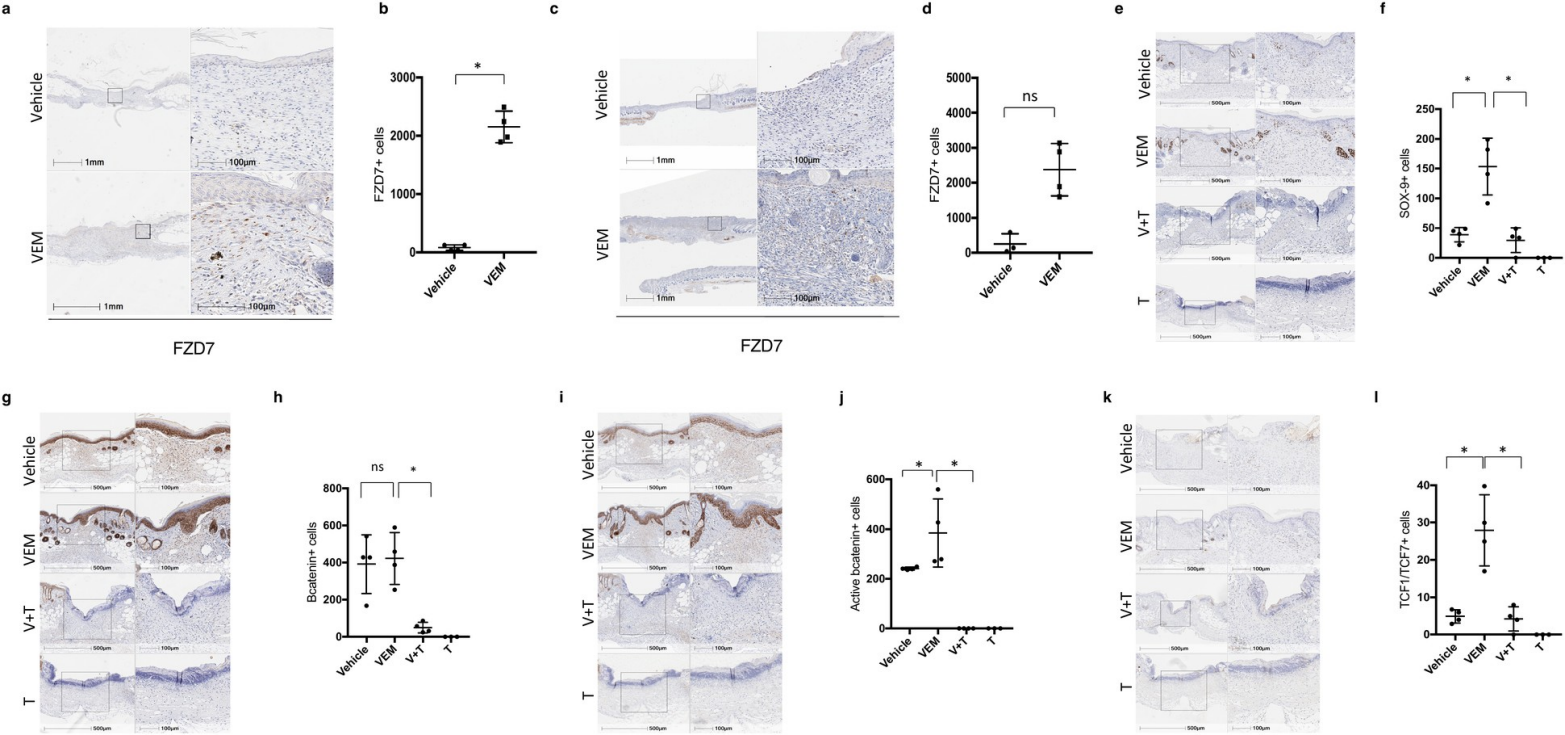
Topical vemurafenib enhances FZD7 and promotes β-catenin signaling which is reversed by MEK inhibition. (a and c) Representative FZD7 staining IHC images in the presence and absence of vemurafenib in (a) db/db mice and (c) Balb/c wounds by day 16. (b and d) Scatter plots representing the quantification of FZD7+ cells (*P = 0.02, n = 4) for control wounds versus treated db/db (b) and Balb/c wounds (d) on day 16. (e) Representative images of Sox-9 IHC staining of vehicle-, vemurafenib-, vemurafenib plus trametinib (T), trametinib alone (T) treated C3H wounds on day 6 (f) Scatter plots representing the quantification of SOX-9+ cells (P = 0.02; Vehicle vs vemurafenib and vemurafenib vs V+T, n = 4) for control wounds versus treated C3H wounds on day 6. (g) Representative images of β-catenin IHC staining of vehicle-, vemurafenib-, vemurafenib plus trametinib (V+T), trametinib alone (T)- treated C3H wounds on day 6. (h) Scatter plots representing the quantification of β-catenin+ cells (*P = 0.02, vemurafenib vs V+T, n = 4; ns, vehicle vs vemurafenib and vemurafenib vs T) for control wounds versus treated C3H wounds on day 6. (i) Representative images of active β-catenin IHC staining of vehicle-, vemurafenib-, vemurafenib plus trametinib (V+T), trametinib alone (T)- treated C3H wounds on day 6. (j) Scatter plots representing the quantification of active β-catenin + cells (*P = 0.02; Vehicle vs vemurafenib and vemurafenib vs V+T; n = 4) for control wounds versus treated C3H wounds on day 6. (k) Representative images of TCF1/TCF7 IHC staining of vehicle-, vemurafenib-, vemurafenib plus trametinib (T), trametinib (T)- treated wounds on day 6. (l) Scatter plots representing the quantification of TCF1/TCF7+ cells (*P = 0.02; vehicle vs vemurafenib and vemurafenib vs V+T; n = 4) for control wounds versus treated C3H wounds on day 6. Error bars in b, d, f, h, j and l mean ± s.d. Bar graphs represent one experiment with 3–4 replicates per group.

We next wished to evaluate the mechanism by which vemurafenib induces hair follicle regeneration. In examining the transcriptomics data from the db/db wounds, *Fzd7* transcripts were elevated, but did not reach significance (S1 Table). *Fzd7* encodes the Frizzled-7 protein, a Wnt receptor found in skin. Since the Wnt-β-catenin axis plays a critical role in hair follicle morphogenesis [26–28] and in wound induced hair neogenesis [29, 30], we wished to examine whether activation of the Wnt pathway following topical vemurafenib application could explain the hair follicle regeneration we observed. First, we explored whether *Fzd7* was expressed at higher levels in keratinocytes, together with pErk (S1 Fig), and then we examine Fzd7 levels in mouse wounds. We found that vemurafenib-treated diabetic wounds showed significantly higher Frizzled-7^+^ (FZD7) cells on day 16, compared to control wounds (p = 0.02 by t test, n = 4; Fig 5A and 5B). The expression of the FZD7 was also higher in the non-diabetic (Balb/c) mice vemurafenib-treated wounds (Fig 5C and 5D).

**Fig 6:**
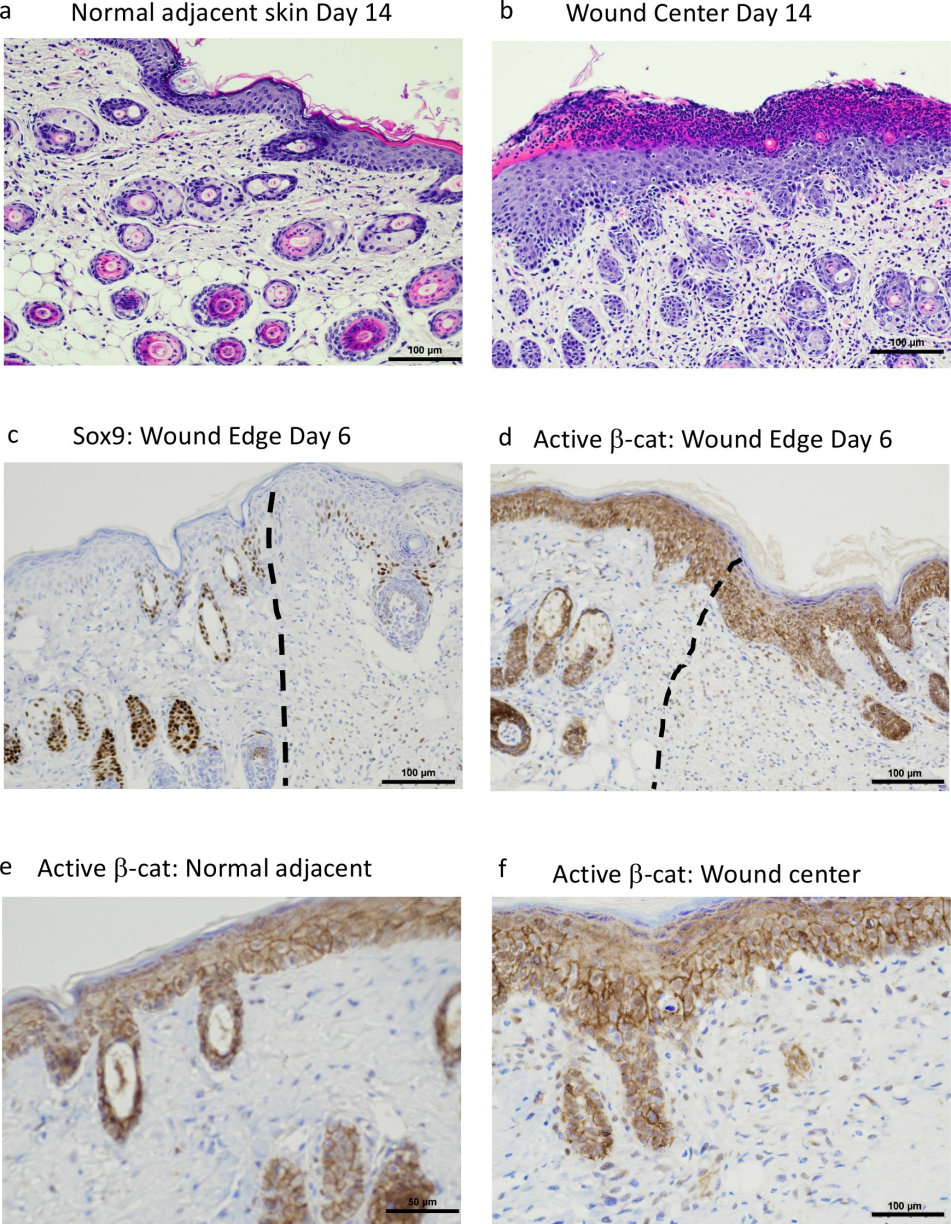
Evidence of hair follicle neogenesis. (a and b) Skin adjacent to a wound demonstrates growth of normal hair follicles and sebaceous glands, embedding in the subcutaneous adipose tissue whereas skin in the center of the wound demonstrates regeneration of many hair follicles, some with sebaceous glands with random orientation in the dermis, directly underlying a hyperplastic, recently reepithelialized epidermis. (c and d) Sox9 and active β-catenin immunohistochemistry showed adjacent skin with typical appearance of hair follicles (left of dotted lines) and wounded tissue (right of dotted lines) with atypical hair follicles and germs expressing Sox9 and active β-catenin in different orientation from normal hair follicles. (e and f) High power view showing the distinct appearance of hair follicles in normal adjacent skin (e) and within the center of a wound (f).

Since *Fzd7* is a receptor for Wnt ligands, this suggests that cells within wounds could result in increased Wnt activation, as Wnt activation is necessary for new hair follicle formation in regenerating wounds [29]. To test this, we performed immunohistochemistry on C3H mice that were given incisional wounds early (6 days) after wounding with the goal of determining whether early hair follicles and/or buds would develop in the presence of paradoxical BRAF activation. Wounds were treated topically with DMSO, vemurafenib, and/or trametinib, a small molecule that inhibits MEK activation downstream of BRAF [31]. The incisional wounds were approximated following incision for 2 days. This model was chosen to evaluate whether early budding of hair follicle neogenesis could be induced by vemurafenib. Indeed, we observed early invaginations of the epidermis consistent with hair follicle buds forming in vemurafenib-treated but not DMSO-treated wounds on Day 6 (Fig 5E, 5G, 5I and 5K). These hair buds occurred both at the center and edge of wounds and were histologically different from the hair follicles of the adjacent normal skin (Fig 6A and 6B), displayed Sox9 staining (Figs 5E, 5F and 6C), and displayed similar expression of total β-catenin as healing wounds (Fig 5G and 5H). We next examined activated β-catenin (Fig 5I and 5J) and Tcf1/7 (Fig 5K and 5L) and found that vemurafenib increased activated β-catenin and Tcf1/7 [32] in wounds, confirming increased β-catenin activation. High power images confirmed the presence of active β-catenin in hair follicle buds forming within the wound beds versus normal adjacent skin (Fig 6D–6F). However, wounds treated with trametinib alone, or vemurafenib plus trametinib demonstrated poor healing, and trametinib prevented the appearance of epithelial buds or Sox9-expressing keratinocytes, confirming decreased ability to form early hair buds. In addition, total β-catenin (Fig 5G and 5H), activated β-catenin (Fig 5I and 5J), and Tcf1/7 (Fig 5K and 5L) were all decreased below levels observed in DMSO-treated wounds, suggesting that MEK activation is required for β-catenin activation in the wound environment. Interestingly, the genes that are upregulated by vemurafenib treatment in the RNAseq data of the db/db model were also enriched for genes near Tcf7 ChIP-seq peaks as recorded in the ChEA 2016 database of ENRICHR (adjusted p = 0.02, see S1G Table). Taken together, these data highlight that BRAF activation by topical vemurafenib can trigger β-catenin activation and early hair neogenesis through activation of MEK.

## Discussion

Wound repair is largely skewed to scar formation over regeneration. The goal of regenerative medicine is to restore functional tissue after injury. This is of critical importance in diabetic foot ulcers, as these wounds not only heal slowly, but the tissue that forms is often weak and is prone to reinjury, often resulting in amputation. In this study, we extend our previous findings [12] to demonstrate that paradoxical MAPK activation by topical BRAF inhibition not only accelerates wound healing in the impaired db/db model of diabetic wound healing, but unexpectedly induces regenerative healing with new hair follicle formation in diabetic mouse wounds and two other mouse models. Taken together with our previous findings that the tensile strength of healed tissue in the presence of vemurafenib is higher than that of control wounds [12], our findings highlight the regenerative potential of a simple, topical approach without additional stem cells, growth factors, or extracellular matrices.

While human wounds typically heal with scar formation, large mouse wounds have demonstrated an ability to heal regeneratively as new, disorganized hair follicles throughout the wound in a process termed hair follicle neogenesis [29]. This process is complex and involves multiple cell types including keratinocytes, dermal papillae, dermal adipocytes, fibroblasts, and immune signals [30, 33–35]. Typically, small wounds on mice do not develop significant regenerative capacity and instead heal with a scar. More recently, small wounds have shown the ability to develop new hair follicles through genetic activation of sonic hedgehog and Wnt signaling [15]. Using the small murine wound model, we found that topical vemurafenib activation can provide pro-regenerative signals resulting in new hair follicle formation in three different mouse strains (db/db, Balb/c, and C3H). In our studies, the hair follicles seen within the wounds expressed the stem cell markers Sox9, Krt15, but were surrounded by CD34+ stromal cells. and were histologically distinct from the hair follicles of the surrounding tissue, both in direction of orientation and embedding location in the mid dermis of the wound bed rather than the typical location in the subcutis. Hair buds were forming as early as day 6 following incisional wounds. These findings suggest these hairs as neogenic ones rather than pre-existing hairs. It is important to point out that CD34+ cells were surrounding the collections of Krt15+ cells, so the staining pattern is not consistent with a terminal telogen hair, but further studies are needed to confirm the origin of those hairs [36–39]. In addition, blockade of MEK with trametinib, can prevent hair follicle formation and can override the appearance of hair follicles within wounds induced by BRAF activation, suggesting that intact BRAF-MEK signaling is required for vemurafenib to induce regenerative healing.

Further, we provided evidence for a direct molecular link between BRAF/MEK/ERK activation and β-catenin activation, a pathway critical for hair morphogenesis and hair neogenesis [40]. Paradoxical BRAF activation by vemurafenib results in enhanced β-catenin signaling in the epidermis as evidenced by active β-catenin and TCF1/7 but is completely blocked by downstream MEK inhibition with trametinib.

Collectively, these studies demonstrated that topically applied BRAF inhibitors may be used in impaired wounds, such as diabetic foot ulcers, to enhance skin wound healing through paradoxical BRAF activation. We further identified the potential of BRAF inhibitors to induce regenerative healing by activating β-catenin signaling to induce hair follicle formation. The phenomenon of hair follicles observed within vemurafenib treated wounds could represent 1) wound induced hair anagen growth (WIH-A), neogenic hair follicles similar to the model of wound induced hair neogenesis (WIHN) or an entirely different process all together. Any of these phenomena are suggestive of a positive change to the wound architecture of typical scars that lack adnexal structures, and we believe it further investigation is warranted to determine the origin of these hair follicles. Some features that are observed in WIHN and WIH-A were present in our samples, but not all [29, 41]. This is likely because we did not use the classical "large wound" model that is used to generate WIHN. While some samples displayed hair buds within the center of the wound area, the majority of the hairs we observed were found towards the periphery of the wounds where WIHN does not occur, but where WIH-A occurs [41]. Since the hair epithelium stained with K15 and Sox9, but did not stain with CD34, a marker of a hair follicle in anagen [37]. The CD34 staining was found in a stromal cells surrounding the hair follicle. While the origin of these hair follicle structures is not entirely certain, it is clear that this phenotype depends on activation of the MAPK pathway, as MEKi samples did not display any such hair follicle structures at the center or periphery of wounds but further work is needed to better characterize the tissue phenotype within Brafi treated wounds. Further studies will be directed at identifying endogenous signals that can promote BRAF-MEK activation in wounds to promote regenerative healing.

## Supporting information

S1 Fig Topical vemurafenib upregulates pErk and FZD7 in keratinocytes. (a) Western blot analyses of pERK, total ERK, FZD7 and GAPDH in human epidermal adult keratinocytes treated with vehicle or vemurafenib (1.5 μM).
(TIF)

S1 Table Full list of genes differentially expressed by 1.5-fold or more in either the vemurafenib-treated or control wounds on day 16. (A) normalized gene expression, (B) differential expression between the vemurafenib-treated and control wounds on day 16, and (C, D, E, F, G) ENRICHR-based enrichment analysis in the respective gene sets/gene ontology/pathway on genes with at least 1.5-fold expression change in Table B.
(XLSX)

S1 Raw images (PDF)
