# Supplementary figures and images for: Wound healing with topical BRAF inhibitor therapy in a diabetic model suggests tissue regenerative effects
Source: PLoS One. 2021 Jun 23;16(6):e0252597. doi: 10.1371/journal.pone.0252597 (PMC8221471; doi:10.1371/journal.pone.0252597)

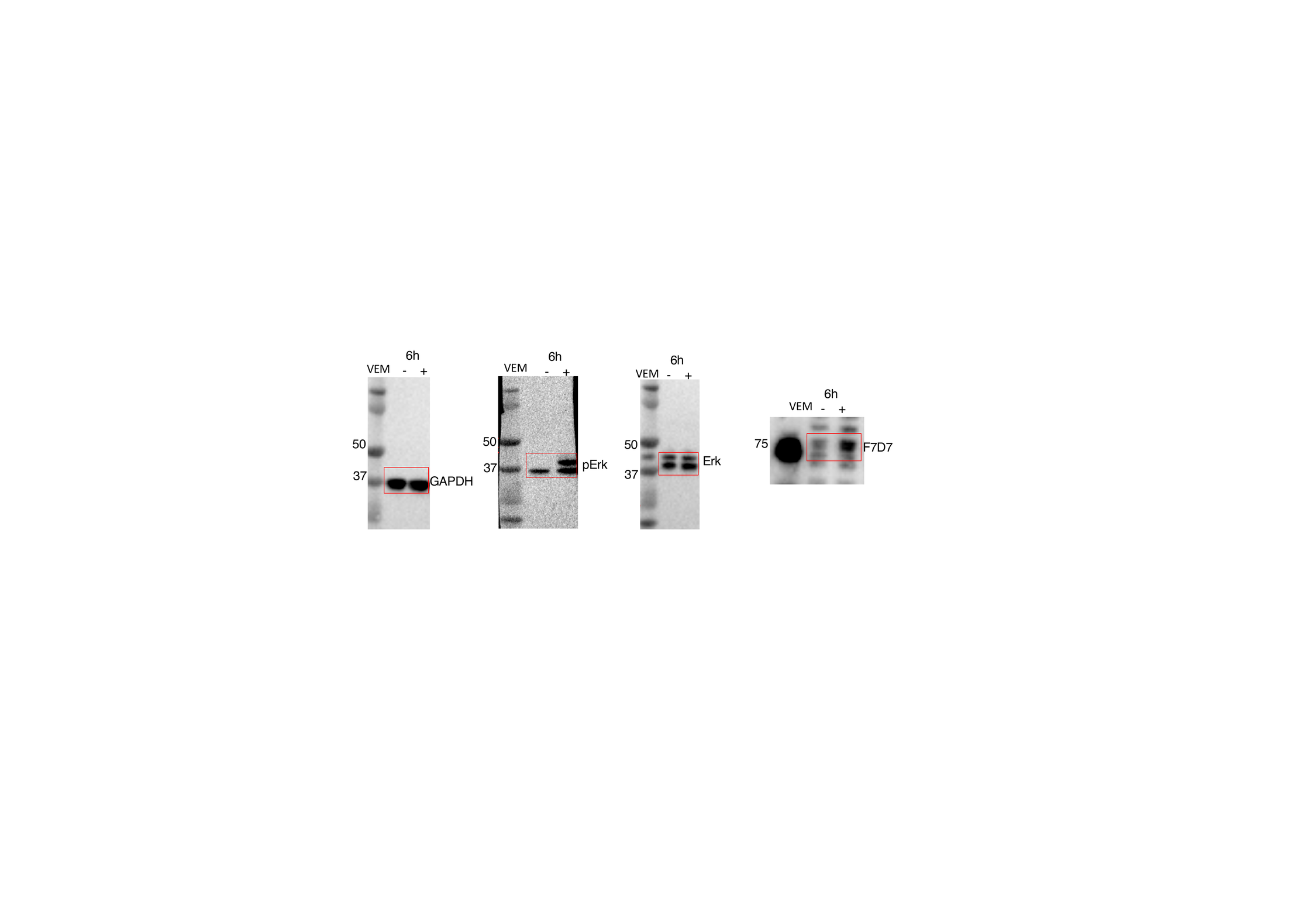

Supplement: S1 Fig — (a) Western blot analyses of pERK, total ERK, FZD7 and GAPDH in human epidermal adult keratinocytes treated with vehicle or vemurafenib (1.5 μM). (TIF) [file pone.0252597.s001.tif]

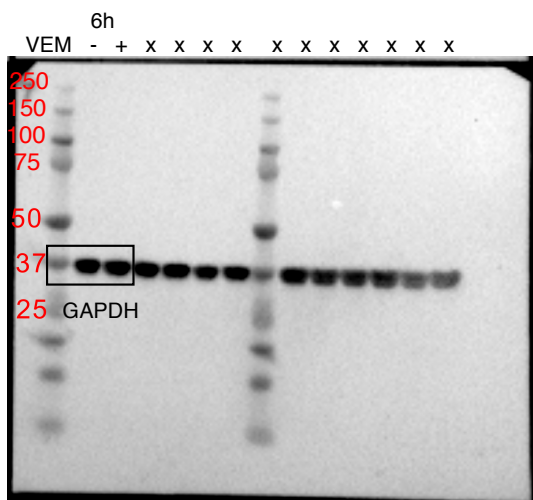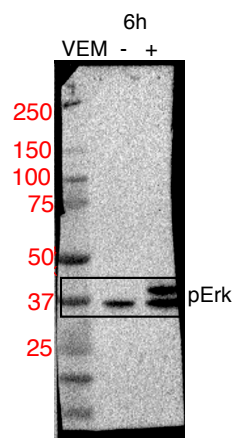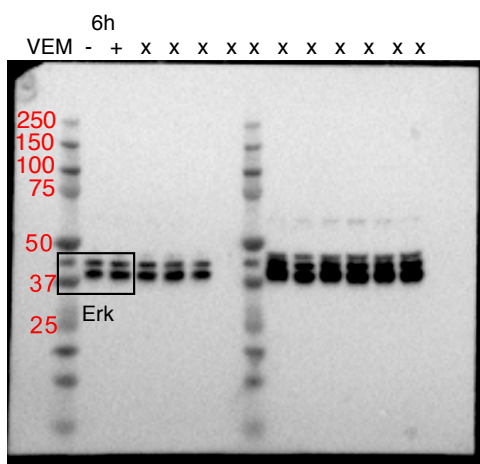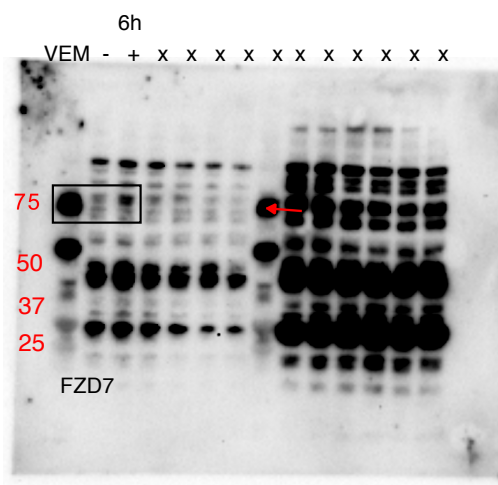

Supplement: S1 Raw images — (PDF) [file pone.0252597.s003.pdf]
